# Effective method for drug injection into subcutaneous tissue

**DOI:** 10.1038/s41598-017-10110-w

**Published:** 2017-08-29

**Authors:** Hyejeong Kim, Hanwook Park, Sang Joon Lee

**Affiliations:** Department of Mechanical Engineering, Pohang University of Science and Technology, Pohang, 37673 Gyeongsangbuk Republic of Korea

## Abstract

Subcutaneous injection of drug solution is widely used for continuous and low dose drug treatment. Although the drug injections have been administered for a long time, challenges in the design of injection devices are still needed to minimize the variability, pain, or skin disorder by repeated drug injections. To avoid these adverse effects, systematic study on the effects of injection conditions should be conducted to improve the predictability of drug effect. Here, the effects of injection conditions on the drug permeation in tissues were investigated using X-ray imaging technique which provides real-time images of drug permeation with high spatial resolution. The shape and concentration distribution of the injected drug solution in the porcine subcutaneous and muscle tissues are visualized. Dynamic movements of the wetting front (WF) and temporal variations of water contents in the two tissues are quantitatively analyzed. Based on the quantitative analysis of the experimental data, the permeability of drug solution through the tissues are estimated according to permeation direction, injection speed, and tissue. The present results would be helpful for improving the performance of drug injection devices and for predicting the drug efficacy in tissues using biomedical simulation.

## Introduction

**Figure 1:**
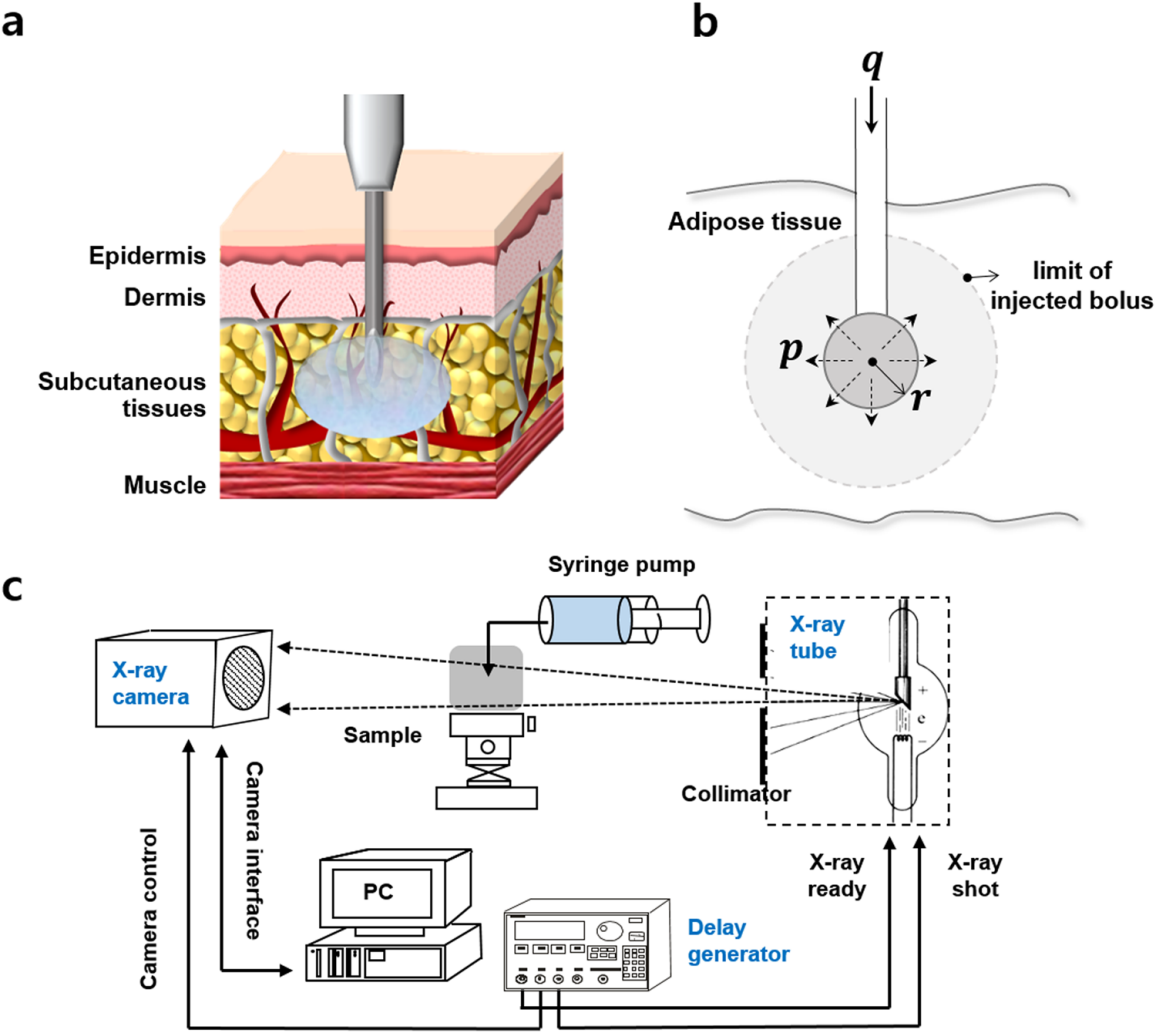
The diagrammatic representation of subcutaneous drug injection and analysis. (**a**) Subcutaneous drug injection beneath the skin. (**b**) Schematic model of subcutaneous injection with assuming that the drug forms a depot of spherical shape. (**c**) The experimental setup to visualize the effects of drug injection using X-ray imaging technique.

Drug injection is a favored method for delivering a drug to get desired effects quickly and directly. Among the different drug injection methods, subcutaneous injection is the one which is applied to the fatty layer of subcutaneous tissue just beneath the skin (Fig. 1a). As subcutaneous tissue has few blood vessels, the injected drug is diffused very slowly at a sustained rate of absorption. Therefore, it is highly effective in administering vaccines, growth hormones, and insulin, which require continuous delivery at a low dose rate. For some pain medications, such as morphine and hydromorphone and allergic medications, the subcutaneous tissue injections are the preferred choice. These drugs are administered by one-shot injection by a syringe or by long-term injection using a pump installed on the skin surface. For instance, automatic injection of epinephrine is used to quickly treat severe allergic reactions^1^. Insulin is administered both by syringe and by insulin pump at a low dose rate to spread the drug over a long period of time^2–5^. The slow injection rate resembles the situation at which insulin is gradually released from the pancreas.

Although drug injections have been administered in a similar manner for a long time, the guideline for drug injection has been continuously updated based on clinical trials for better treatment with overcoming technical limitations^1, 2, 6–9^. The most dominant limitation is considerable variation in the absorption and action of drug from patient to patient, but more importantly from injection to injection for the same patient^10–13^. Thus, patients cannot be certain to experience the same drug effect with similar injections. For instance, the variability in insulin absorption was reported as 15~25% for the same patient, and as 20~45% among patients^11^. Another limitation is discomfort and pain associated with repeated drug injections, which would make patients skip or in worst case he or she may cease the clinical treatment^14^. This might be particularly important for patients with a chronic disease that requires life-long sustained treatment, such as insulin supply to diabetic patients. Although the injection needles and pumps used for drug injection have been improved, patients still have the fear of injection pain and needle-phobia^15, 16^. In addition, frequent subcutaneous injections may lead to skin disorders, such as lipohypertrophy and lipoatrophy, where extra subcutaneous tissue is accumulated under the skin or the size of adipocytes is decreased around the injection site.

To avoid these side effects of frequent subcutaneous injection, injection devices should be designed based on dose effects of drug on tissues to minimize the variability and pain of injection or to reduce the number of injections to patients. Therefore, it is important to exactly understand the effects of injection factors that influence the permeation and diffusion of the drug in subcutaneous tissue. In previous studies, the effects of injection conditions, such as needle length, injection volume, viscosity, and flow rate on depot formation and pain were investigated^17, 18^. However, it is still difficult to select the ideal injection condition for reducing the variability and pain during injecting the drug^14, 19^. Therefore, a more systematic study on the effects of injection condition should be conducted to discriminate the injection factors with respect to the efficacy of drug and to improve the efficacy of the drug^12^.

In previous studies, the formation of drug depots has been investigated using histological study, X-ray computed tomography^10, 17, 20^, radioactive labeling^21, 22^, and ultrasound measurement^8, 23^. However, these methods are time consuming and the test samples should be prepared by histological sectioning or by cryo-freezing. In addition, they have technical limitations in terms of non-invasive measurement and spatial and temporal resolution to investigate dynamic behaviors of the injected drug in subcutaneous tissue. Therefore, an advanced time-resolved, non-invasive bio-imaging technique is required to measure temporal variations of the permeation and diffusion phenomena of drug solution in subcutaneous tissue.

To overcome these technical limitations, we investigated the effects of injection conditions on tissues using real time X-ray imaging technique (Fig. 1c). 2D X-ray imaging technique was employed to visualize the shape and spatial concentration profile of the injected drug solution. This imaging technique provides information on the temporal variation of solution thickness averaged in the direction of X-ray propagation with high spatial resolution. Dynamic movements of wetting front (WF) and variations in water contents in the subcutaneous tissues were quantitatively analyzed as a function of time from the initial impingement to the final saturation. Drug injections were performed in porcine subcutaneous tissues and muscle tissues under *ex vivo* condition. Porcine tissue was selected as a suitable test model, because its subcutaneous fat layer has similar morphological structures and mechanical properties as human tissue^24–26^. The test procedures include single-shot injection with a syringe pump and long-term or prolonged injection using an automatic pump. The present investigation contains an important information for improving the performance of injection devices and for predicting the injection effect of certain target drug. Moreover, the results imply that the X-ray imaging system with high spatial resolution has strong potential in the pharmaceutical analysis of drug injection.

## Results and Discussion

### Permeability of the test tissue

The transport of a drug solution through a tissue, which can be considered as a porous medium, is assumed to follow the Navier-Stokes equation. The tissue can be assumed to be homogeneous, and drug spreads symmetrically in a spherical shape around the injection site. The flow of the injected drug solution is assumed to be stationary and incompressible, where the viscous drag is proportional to flow velocity. Then, the injected flow can be simplified to follow the Darcy’s law;

1 $$v=-k\nabla p,$$

where *v* is the flow rate through the unit cross sectional area, *k* is the hydraulic permeability of the tissue, and *p* is the total pressure acting on the drug solution. The *k* is determined by the interaction between the drug solution and the material property of the tissue, and *p* is determined by the injection speed of drug solution.

A drug solution is injected at a volumetric flow rate *q*, into subcutaneous tissue. Assume that the fluid fills a reservoir of radius *r*, and pressure *p*_*w*_ is formed at the tip of the needle, and the surrounding tissue has a permeability *k* (Fig. 1b). The volumetric flow rate *q*, is related to the flow rate per unit area, *v*, according to

2 $$v(r)=\frac{q}{4\pi {r}^{2}}.$$

Substituting the equation (2) into the equation (1) gives

3 $$-k\frac{\partial p}{\partial r}=\frac{q}{4\pi {r}^{2}}.$$

By integrating equation (3) with respect to *r*, the pressure *p*_*t*_ with respect to the injected bolus radius *r*_*t*_ at time *t* is

4 $${p}_{t}=\frac{q}{4\pi k{r}_{t}}.$$

The pressure acting on a unit surface area of the tissue *p*, is estimated as^27^

5 $$p[kPa]=0.74\times q+23.$$

Then, the permeability *k* can be expressed as follows;

6 $$k=\frac{1}{2.96\pi \cdot {r}_{t}}\cdot (\frac{q}{q+31.08}).$$

Based on the experimental data of wetting front measurements, the permeability *k* values of the subcutaneous and muscle tissues at different flow rates and movement directions were estimated (Table 1). The estimated value at a slow injection rate of 25 *μL/min* ~ 100 *μL/min* is $1.07 \sim 4.41\times {10}^{-13}{m}^{4}/N\cdot s$. It is in the same order of magnitude with the reported values ranged from $1.8\times {10}^{-12}\,{\rm{to}}\,1\times {10}^{-13}{m}^{4}/N\cdot s$ ^28, 29^.

**Table 1:** The estimated permeability values in the subcutaneous and muscle tissues at different flow rates and diffusion directions.

Tissue	Flow rate (*mL/min*)	Permeability (*m* ^4^/*N* · *s*)	
		**Horizontal**	**Vertical**
Subcutaneous tissue	0.025	1.07 × 10^−13^	1.38 × 10^−13^
	0.1	1.07 × 10^−13^	4.41 × 10^−13^
	6	7.27 × 10^−12^	1.27 × 10^−11^
Muscle tissue	6	5.47 × 10^−12^	1.27 × 10^−11^

### Slow injection of drugs on subcutaneous tissues

**Figure 2:**
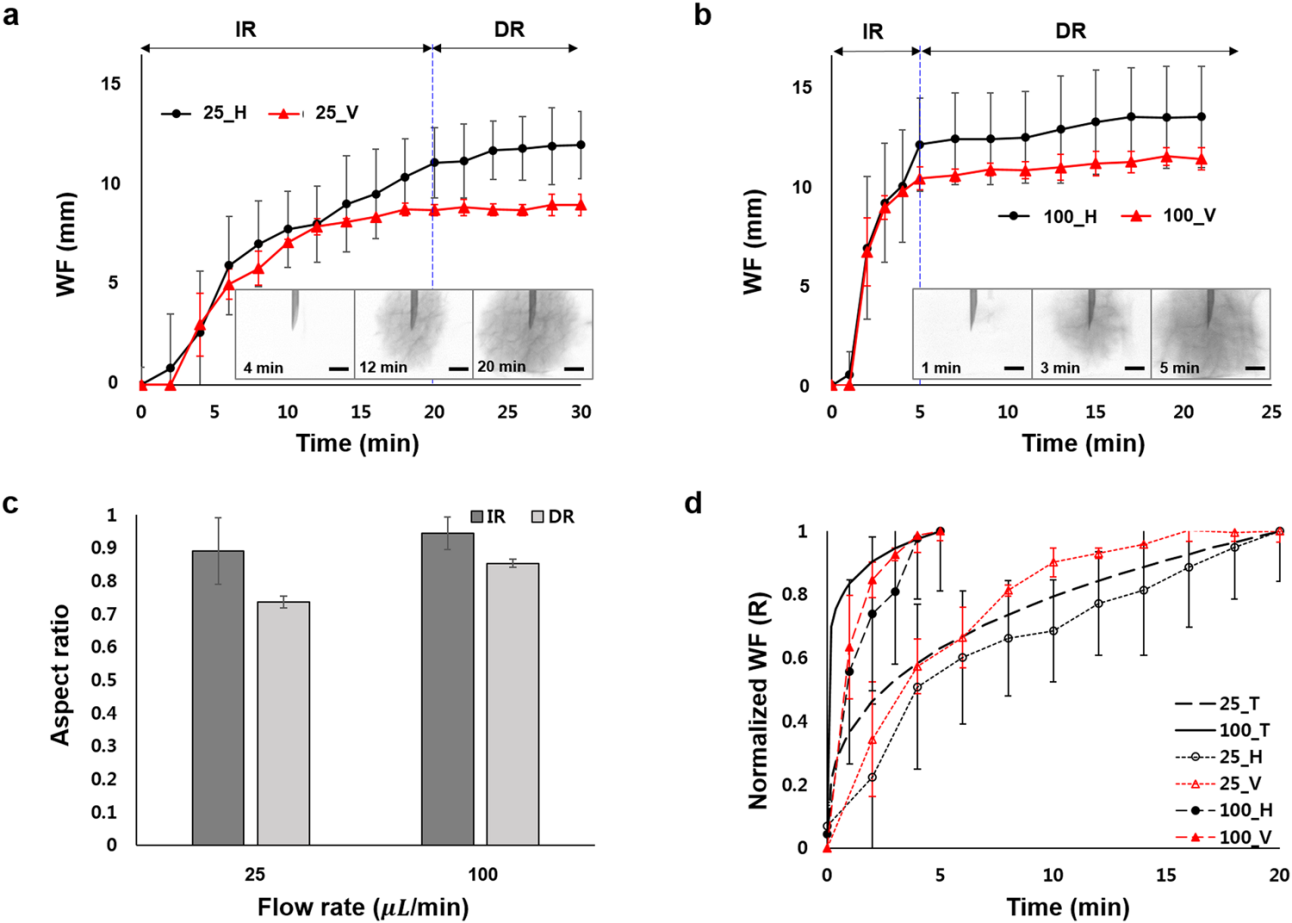
Effect of injection rate on spreading of drug solution. Temporal variations of the WF position through the tissue when the drug solution was injected at volume infusion rates of (**a**) 25 *μL/min* and (**b**) 100 *μL/min*. Insets are X-ray images of the injection region of the subcutaneous tissues. (**c**) Aspect ratios of depot formations in the injection and diffusion regions. (**d**) Comparison of the experimental results and theoretical predictions of normalized WF. Scale bar: 500 *μm*.

Analogous to the subcutaneous injection using an automatic pump, where the dose is spread over a long period of time, the effect of the slow injection rate was investigated. The total injection volume of drug solution was tuned as 500 *μL*, and the solution was administered at two different injection rates of 25 *μL/min* and 100 *μL/min*. Once the drug solution starts to perfuse into the tissue, it preferentially passes along the intercellular area in the tissues which have a typical alignment pattern (Fig. 2a and b, insets). Depending on the alignment pattern of the tissue, different depot of the injected drug solution has formed.

The temporal variations of WF position in the test tissues are investigated (Fig. 2a and b). The WF information is classified into two regions viz. injection region (IR) and diffusion region (DR). In the IR, the injection pressure is dominant compared to other forces such as gravity and capillary, and the permeation of drug solution can be explained by the Darcy’s law of equation (1). In the DR, however, the solution is diffused by concentration gradient, and the WF does not increase dramatically, irrespective of injection speed.

At the initial stage in the IR, the propagation velocities in the horizontal and vertical directions are similar in both injection cases. However, at the last stage, the solution propagates faster along the horizontal direction than the vertical direction. For quantitative analysis, the aspect ratio of the depot formation is considered as WF_v_/WF_h_, where WF_v_ and WF_h_ indicate the WF in the vertical and horizontal directions, respectively (Fig. 2c). In the IR, the aspect ratios of depot formation are 0.89 ± 0.1 and 0.94 ± 0.05 at 25 *μL/min* and 100 *μL/min* injection rates, respectively. As the aspect ratios approach are almost near 1, the depot formation can be assumed to have a spherical shape. In the DR, the corresponding aspect ratios are 0.73 ± 0.02 and 0.85 ± 0.01 at 25 *μL/min* and 100 *μL/min* injection rates, respectively. This indicates that the drug solution diffuses faster along the horizontal direction, compared to the vertical direction. Due to typical alignment of the tissue, the solution preferentially diffuses faster through the wider intercellular area^17, 20^. The subcutaneous tissue seems to have wider intercellular area along the horizontal direction, which gives rise to faster diffusion of the drug solution.

With the assumption of spherical depot formation in the IR, the temporal variation of WF(t) has the following relationship with flow rate q;

7 $$\varepsilon \cdot \frac{4}{3}\pi \cdot {\rm{WF}}{({\rm{t}})}^{3}=q\cdot t.$$

where *ε* is the effective volume fraction of the solution filled in the IR, and t is the injection time. Then, the WF can be expressed as

8 $$WF({\rm{t}})=\sqrt[3]{\frac{3\cdot q\cdot t}{4\pi \varepsilon }}.$$

By dividing equation (8) with the maximum radius $W{F}_{m}=\sqrt[3]{\frac{3\cdot Q}{4\pi \varepsilon }}$, where Q is the total injection bolus, the normalized WF, R, is obtained as follows;

9 $${\rm{R}}({\rm{t}})=\frac{{\rm{WF}}({\rm{t}})}{W{F}_{m}}=\sqrt[3]{\frac{q\cdot t}{Q}}.$$

The experimental results and theoretical prediction of normalized WF were compared and showed in Fig. 2d. The experimental results were well corresponded to the theoretical predictions with R^2^-values of 0.9 ± 0.03 and 0.86 ± 0.11 for injection rates of 25 *μL/min* and 100 *μL/min*, respectively.

**Figure 3:**
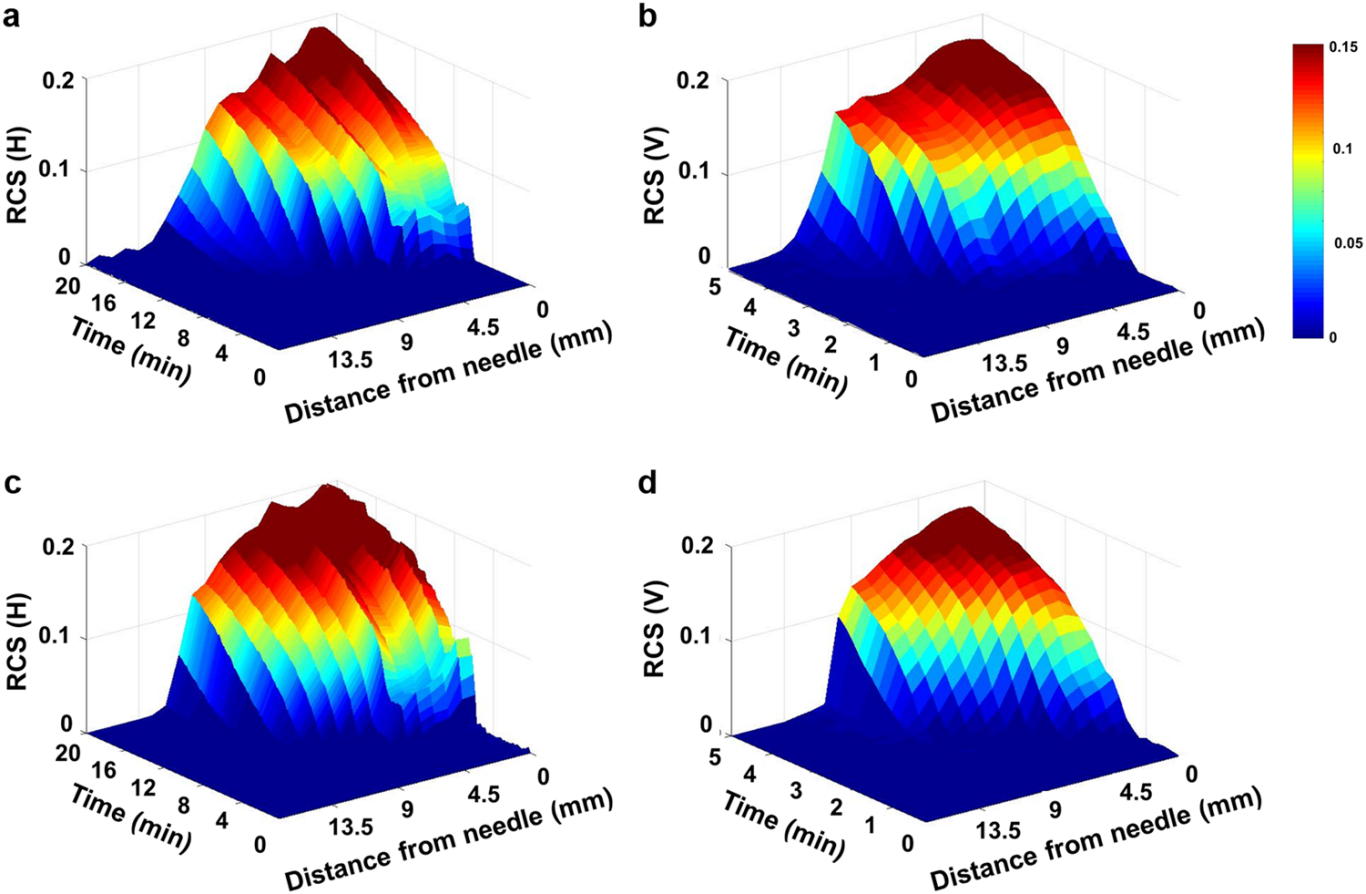
Temporal evolutions of the (**a**) horizontal and (**b**) vertical RCS values at the injection rates of 25 *μL/min*. Temporal evolutions of the (**c**) horizontal and (**d**) vertical RCS values of the injection rate of 100 *μL/min*.

**Figure 4:**
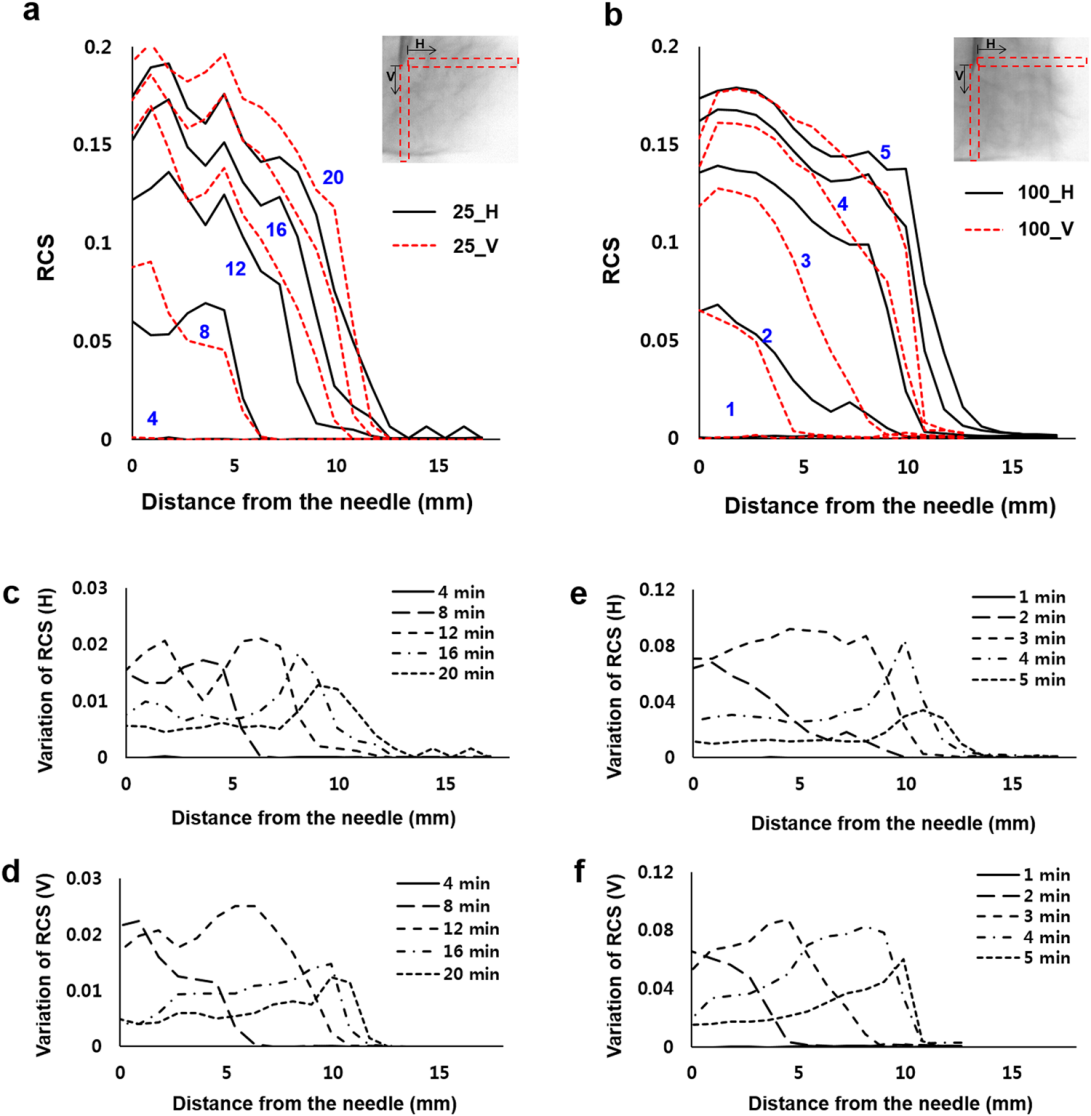
RCS distribution was quantitatively analyzed according to the distance from the needle. Variations of the normalized RCS values along the horizontal and vertical directions at the volume infusion rates of (**a**) 25 *μL/min* and (**b**) 100 *μL/min*. Temporal variations of RCS along the (**c**) horizontal and (**d**) vertical directions of the volume infusion rate of 25 *μL/min*. Temporal variations of RCS values along (**e**) horizontal and (**f**) vertical directions at the injection rate of 100 *μL/min*.

The Fig. 3 shows the relative content of the solution (RCS) from the needle in the direction of X-ray propagation. Figure 3a and b show temporal evolutions of the horizontal and vertical RCS values at the injection rate of 25 *μL/min* whereas, Fig. 3c and d represent the temporal variations of the horizontal and vertical RCS at the injection rate of 100 *μL/min*. In both the cases the drug solution spreads wider along the horizontal direction than the vertical direction. For better comparison, the RCS distributions were quantitatively analyzed through the distance from the needle of injector (Fig. 4). Based on the attenuation of X-ray absorption rate, the spatial distributions of the drug solution were analyzed quantitatively. The intensity values in the test sample of 0.9 *mm* × 0.9 *mm* are averaged and normalized along the horizontal and vertical directions through the distance from the needle (Fig. 4a and b, insets, red dotted boxes). At both injection rates, the variations of RCS values along the horizontal and vertical directions exhibit similar tendency. The RCSs have the maximum values in the region just near the needle. With getting away from the needle, the RCS values are gradually decreased. The decreasing slope of the RCS values near the WF, which indicates the concentration gradient of the drug solution, increases with increase in time.

The temporal variations of RCS, which is determined as RCS variation per time interval,

10 $$Variations\,of\,RCS=\frac{RCS({t}_{2})-RCS({t}_{1})}{{t}_{2}-{t}_{1}}$$

were analyzed in the horizontal and vertical directions (Fig. 4c–f). Figure 4c and d show the results at the injection rate of 25 *μL/min* and Fig. 4e and f at the injection rate of 100 *μL/min*. For both cases, as time passes the locations of the maximum RCS values move away from the site of needle injection. This indicates that the solution gradually moves away from the needle with filling the tissue. In addition, all of the RCS variations have positive values. This implies that although the tissue near the needle is not saturated with the solution, the injected solution permeates further outward. The RCS values have several peaks with large variations which is due to the presence of the aligned intercellular area through which the drug solution preferentially permeates.

When the solution injected slowly at a flow rate of 25 *μL/min* and it was visualized, the RCS does not increase up to the initial 220 s after injection. At 100 *μL/min*, the solution starts to be appeared after 60 s of injection, which is almost 4 times faster than that of 25 *μL/min*. The delay of effective drug injection into the target tissue implies that the injection rate of drug solution should be increased to provide additional force to penetrate the tissue to inject drug. Once the injection force was enough to overcome the hydraulic resistance of the tissue, the accumulated drug solution was abruptly injected into the tissue. To ensure the injection of the full dose, the force delivered by the injection device must overcome the pressure losses encountered in the injection process, and the tissue resistance pressure (TRP)^30^. The TRP has the following relationship with the infusion rate *q*; $TRP=10.4\times q+1.14$ ^3^. Based on this relationship, the TRP is 1.4 *kPa* and 2.2 *kPa* at injection rates of 25 *μL/min* and 100 *μL/min*, respectively. As the drug solution is accumulated at the tip of the injection needle, the pressure is increased up to the threshold pressure to overcome the hydraulic resistance of the tissue. This may induce the delay of drug injection into the tissue.

### Fast injection of drugs on different tissues

Analogous to the single-shot injection by a syringe pump, the effects of injection location on drug diffusion in the tissue were investigated. The drug solution was injected into the subcutaneous and muscle tissues, and the variations of WF and RCS after drug injection were compared. An insulin solution of 500 *μL* was injected into the target tissue at a flow rate of 6 *mL*/*min*.

**Figure 5:**
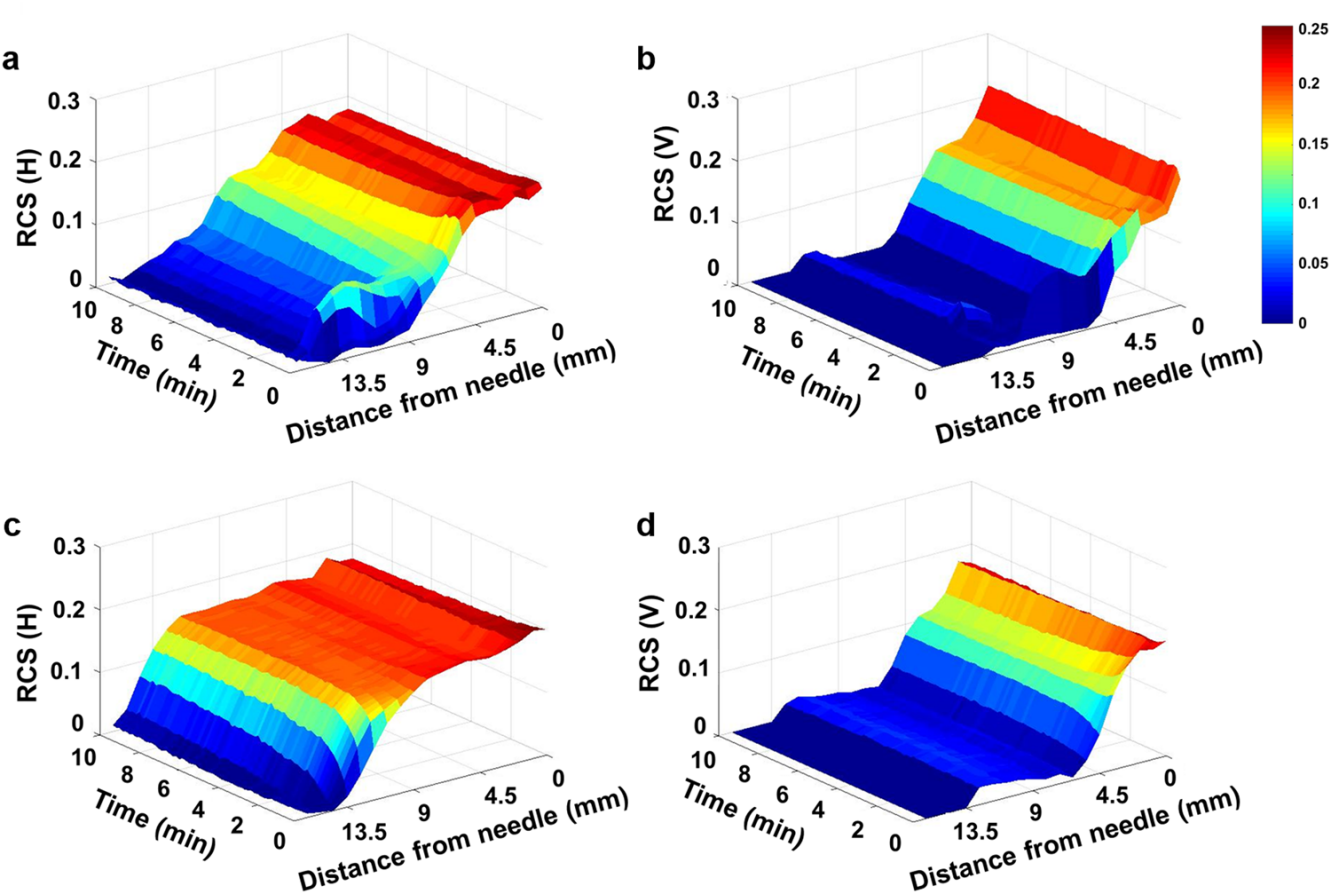
Temporal evolutions of (**a**) horizontal and (**b**) vertical RCS values in the subcutaneous tissue. Temporal evolutions of the (**c**) horizontal and (**d**) vertical RCS values in the muscle tissue.

The Fig. 5 shows variations of the vertical and horizontal RCS in the direction of X-ray propagation. Figure 5a and b show the temporal variations of the horizontal and vertical RCS in the subcutaneous tissue, respectively. Whereas, Fig. 5c and d show the temporal evolution of the horizontal and vertical RCS in the muscle tissue. The drug solution spreads wider along the horizontal direction, compared to the vertical direction for both tissues. Interestingly, the horizontal diffusion of the drug solution injected into the muscle tissue is faster than that in the subcutaneous tissue.

**Figure 6:**
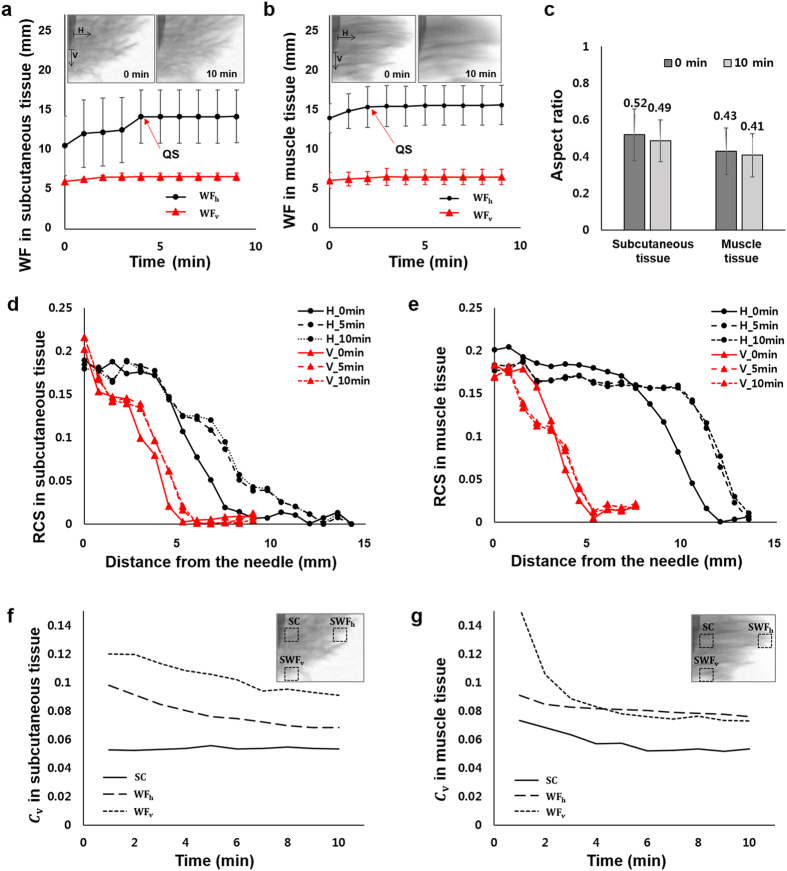
Temporal variations of WF_h_ and WF_v_ in the (**a**) subcutaneous tissue and (**b**) muscle tissue, respectively. Insets are X-ray images of the subcutaneous and muscle tissues at 0 *min* and 10 *min* after drug injection. (**c**) Aspect ratios in the subcutaneous and muscle tissues at 0 *min* and 10 *min* after drug injection. Variations of RCS values according to distance from the needle in (**d**) the subcutaneous tissue and (**e**) the muscle tissue. Temporal variations of C_v_ in the (**f**) subcutaneous tissue and (**g**) muscle tissue, respectively. The C_v_ values are measured at the regions of SC, SWF_h_, and SWF_v_ (insets, dotted boxes). Scale bar: 500 *μm*.

Temporal variations of WF_h_ and WF_v_ at the site of injection are depicted in Fig. 6a and b. Insets show X-ray images of subcutaneous tissue and those of muscle tissues at 5 *min* and 10 *min* after drug injection. Immediately after drug injection, the WF_h_ increases twice than WF_v_ in both subcutaneous and muscle tissues. As the muscle tissue is more permeable than the subcutaneous tissue^17^, the initial WF_h_ in the muscle tissue is wider than that of the subcutaneous tissue when the same injection force is applied. As the time passes, the WF values approach to the quasi-saturation (QS) value. The QS time is 4 *min* and 2 *min* in the subcutaneous and muscle tissues, respectively.

The aspect ratio of the depot formation in the subcutaneous tissue is larger than that in the muscle tissue (Fig. 6c). In the horizontal direction of drug diffusion, more solution permeates in the muscle tissue than that in the subcutaneous tissue. In the case of fast injection, the aspect ratio is dramatically decreased compared to the slow injection where the solution permeates in the horizontal and vertical directions at a similar rate (Fig. 2c). This indicates that the more drug solution permeates in the horizontal direction than the vertical direction when the drug is injected rapidly. This may be attributed to the funneling fracture of the tissue. During the injection process, the solution tunnels through pathways having low hydraulic resistance, which is called as preferential flow, possibly between lobules of adipocytes in the subcutaneous tissue. The fracture toughness, J, related with the tunneling fracture of a micro-crack of width *h* in a tissue of Young’s modulus *E*, under a pressure *P* is given as $J=\frac{{P}^{2}h}{1.27E}$ ^27, 31^. When the injected solution has the higher pressurized flow than the fracture toughness, a connecting network of micro-cracks was created with increasing the permeability of the tissue^26, 32^. The fracture toughness of the subcutaneous tissue is *J*_*a*_ = 4.1 *kJ/m* ^*2*^, when the average width of micro-cracks inside the tissue is assumed as *h* = 800*μm* ^26^. When the flow rate is 25 *μL/min*, pressure of *P* = 23.3 *kPa* was applied to the tissue according to equation (8), and *J* = 340 *J/m* ^*2*^, of which the order of magnitude is lower than *J*_*a*_. This indicates that the slowly-injected drug solution permeates through interfaces in the subcutaneous tissue without creating the funneling fracture of the tissue. Thus, it has a large effect on the permeability of the tissue. On the other hand, when the flow rate is 6 *mL/min*, pressure of *P* = 94 *kPa* is applied to the tissue, and *J* = 5.5 *kJ/m* ^*2*^, which has similar order of magnitude with *J*_*a*_. In this case of fast drug injection, the solution may create fracture in the tissue, which increases the permeability of the tissue. Therefore, as the permeability in the horizontal direction increases significantly, the aspect ratios in the fast injection case were also dramatically decreased compared to the slow injection (Fig. 2c, Table 1).

Variations of the RCS values along the horizontal and vertical directions in the subcutaneous and muscle tissues are depicted in Fig. 6d and e, respectively. Just near the injection needle, the RCS values in the horizontal and vertical directions are nearly similar. While going away from the needle, we found decrease in RCS values. This indicates that the drug solution diffuses outward from the needle. In addition, the horizontal RCS values are much larger than the vertical RCS values in muscle tissues. We also found that in the subcutaneous tissue, the drug solution was simultaneously spreaded out from the needle in the horizontal and vertical directions until the solution becomes saturated. On the other hands, the insulin solution is rapidly saturated in the vertical direction of the muscle tissue as soon as insulin solution is injected. This indicates that the preferential diffusion along certain direction was closely related to directional arrangements of the tissue.

The intensity value of X-ray image in the solution region decreases with the lapse of time, as the solution is transported to the adjacent cells. To quantitatively analyze this intensity variation, the coefficient of variation (C_v_) of the following definition was employed; *C*_*v*_ = σ/μ, where σ is the standard deviation and μ is the average of intensity values of X-ray image, respectively (Fig. 6f and g). It represents the degree of intensity fluctuations of the drug solution in the region of interest (ROI). Insets show typical X-ray images of subcutaneous and muscle tissues, captured immediately after drug injection. The dotted boxes indicate the ROIs where the C_v_ values were measured. Temporal variations of C_v_ values in the ROIs of surrounding center (SC) around the tip of the needle, surrounding horizontal direction WF_h_ (SWF_h_) and surrounding vertical direction WF_v_ (SWF_v_) were measured.

In the subcutaneous tissue, C_v_ values are not significantly varied in the region SC. This indicates that the drug solution homogeneously permeates through gaps of the tissue having a mesh network (Fig. 6f). The C_v_ values at SWF_v_ and SWF_h_ are gradually decreased with a similar tendency as time goes by. This implies that the drug solution gradually diffuses into adjacent cells. On the other hand, the C_v_ values are decreased in muscle tissue in all ROIs of the muscle tissue with the lapse of time, because the drug solution preferentially permeates along the horizontally-aligned muscle tissues, and diffuses into adjacent cells.

Meanwhile, beyond the QS time, the drug solution does not distinctively spread through the tissue. This kind of event was not expected to occur under real clinical situation as the experiment was conducted using *ex vivo* injections. In addition, the tissue sample does not have vascular flow and temperature and pH are lower than those of living tissues. In real clinical conditions, the variations in drug activity are influenced by several factors, including blood flow, temperature, pharmacokinetics and pharmacodynamics of the drug. In order to obtain experimental results with high clinical relevance, *in vivo* animal test should be carried out in the near future.

### Subcutaneous injection of real pharmaceuticals

Pharmaceuticals administered by subcutaneous injection are usually delivered in small volume (less than 2 *mL*). Pharmaceuticals such as insulin, heparin, vaccines and some hormones, are commonly administered by subcutaneous injections^33–36^. Some diseases require frequent injecting of drugs, such as insulin or heparin. In this case, the injection site should be rotated. Typically, diabetic patients pick one region and rotate the injection sites within the region to maintain consistent absorption of insulin day by day. However, the absorption rate of drug varies from site to site. To keep a consistent therapeutic efficacy of drugs regardless of injection conditions at various injected sites, the effects of subcutaneous injection factors on drug spreading in the injection sites should be clearly understood. The perfusional features of the injected drugs in subcutaneous tissues depend on the injection conditions and injected sites. This may be used as a useful reference to update the guideline for subcutaneous injection. Drugs that need to be arrived at the injection sites very quickly, such as allergic drugs of epinephrine^37^, pain medications of morphine and hydromorphone^38^, or drugs preventing nausea and vomiting of metoclopramide or dexamethasone^39, 40^, can also be administered by subcutaneous injection. When a drug is injected at the tissues having low functional capillary density (FCD), the perfusion velocity inside the tissue is slower than the tissues with high FCD^41^. The drug solution injected at a slower rate arrives deeper in the vertical direction. It may reach veins faster by shortening the depth of tissues with low FCD where the drug may be slowly perfused. If rapid perfusion of drug is needed to the tissue with low FCD, slow injection rate of around 100 *μL/min* would be favorably better than the fast single-shot injection at high injection rate of around 6 *mL*/*min*. On the other hand, when the drug is injected at tissues with high FCD, relatively faster single-shot injection is recommended for spreading the drug widely in horizontal direction to cover as many capillaries as possible.

Improper subcutaneous injection of pharmaceuticals may result in side effects, such as bruising, haematoma and pain at the injection site^33^. One of main factors that may affect those side effects is the injection speed. A previous study suggested that slow injection probably induces less pain and bruising, compared to fast injection^34^. The present results support the previous studies which indirectly estimated the effect of injection conditions on the side effects. The fast injection of drugs into subcutaneous tissues widely permeate into the horizontal direction more than vertical direction by forming depots of elliptical shape (Fig. 6a,b). This may cause more bruising on the skin, compared to slow injection which forms depots of spherical shape (Fig. 2a,b). Therefore, slow injection of drugs is better for reducing bruising or haematoma. In addition, pain relief is another important issue in drug injection. Many studies have been conducted to reduce pain especially in childhood^42^. The concept of fracture toughness, *J*, can be used as a guideline for checking the pain coming from side effects of drug injection. When a drug is injected at a slow rate for which the fracture toughness is less than a certain threshold *J*_*a*_, patients may not feel the pain, because the fracture is not so severe. On the other hand, when the drug is injected in a fast manner, the *J* value of fracture toughness is larger than *J*_*a*_ and patients may feel the pain because the fracture in the surrounding tissues is severe. In considering the mechanical properties of subcutaneous tissue, the injection pressure has to be controlled to minimize the patient’s level of pain in the tissue.

## Conclusion

Drug injection is a favored delivery means of drugs to get desired therapeutic effects quickly and directly. The guideline for subcutaneous drug injection has been continuously updated for overcoming its side effects, such as variability or pain. Thus, it is important to understand the effects of injection parameters on the spreading of drug in subcutaneous tissues.

The temporal variations of the permeation of injected insulin solution in two different tissues were experimentally analyzed at different injection speeds using X-ray imaging technique with high spatial resolution. The injection flow rate is an important parameter in depot formation, because it is closely related with variation of mechanical properties of tissues. The diffusion patterns of the injected drug solution were largely influenced by the properties of tissues. Compared with the diffusion tendency in the horizontal direction, the drug solution injected in the muscle tissue is more diffused than in the subcutaneous tissue.

Using the X-ray imaging technique, we systematically investigated the effects of various injection conditions on drug diffusion in tissues. Temporal variations of spatial distribution of drug solution were quantitatively analyzed from X-ray images captured consecutively. The present results would provide important information in the design of a new advanced drug injection device for easy clinical treatment. In technical point of view, the present X-ray imaging facility would be utilized effectively to investigate permeation and diffusion phenomena of different drug solutions in animal disease model for clinical applications.

The special requirements for subcutaneous injection of pharmaceuticals were discussed. The guidelines for subcutaneous injection of drugs such as injection speed and injection sites, were suggested syntagmatically in consideration of FCD and transport characteristics in the tissues. In addition, a proper injection speed was suggested for prevention of bruising and pain as the side effects of subcutaneous injection.

Because the therapeutic effects of drugs are highly associated with perfusion characteristics inside subcutaneous tissues, the present results could be used to build appropriate personalized injection guideline for individual patients. This eventually leads to the patient-tailored treatment. To expand this research to other drugs, the effect time and transport characteristics of the drugs need to be estimated in the same manner. Therefore, this research will be very helpful in the screening and development of new injectable drugs. In addition, this research would provide material properties of subcutaneous tissues, which is essential in the development of a new drug delivery system.

## Materials and Method

### X-ray imaging

The diagrammatic representation of the experimental setup used in the present study was depicted in Fig. 1c. A medical X-ray source (Varian A272) was used and X-ray images were captured by an X-ray CCD camera (Hamamatsu, C9300, Japan). X-ray beam was converted into visible light by passing through a scintillator combined with fiber optic plate attached in front of the CCD camera. The X-ray tube and CCD camera were synchronized using a delay generator. The field of view was 36.0 *mm* × 24.0 *mm* and the effective pixel size was 9 *μm* × 9 *μm*. The exposure time of the X-ray CCD camera was 1 s. The distance between the test sample to the detector and the X-ray source to the detector were 60 *cm* and 100 *cm*, respectively. The X-ray source has energy spectrum ranged from 15 to 140 *kVp* and the optimal conditions for this research were found to be 80 *kVp* and 100 *mA*.

### Sample preparation

Subcutaneous and muscle tissues of porcine were cut into a cube of 4 *cm* × 4 *cm* with thickness of 4 *cm* (Fig. 1a). The solutions injected into tissues were quantitatively visualized by using X-ray imaging system. 75% of insulin solution, which was adjusted to 100 *units/mL* ( = 3.6 *mg/mL*) in PBS, and 25% iodine contrast agent (Ultravist®, Bayer HealthCare Pharmaceuticals Inc.) was prepared as a drug solution. A syringe pump (PHD 2000, Harvard Apparatus, USA) was utilized to inject the drug solution at various flow rates (Table 2). A 23 G needle was used to inject insulin solution from the tip of the needle as a point source. The tip of the needle was inserted 1 *cm* from the tissue surface. All procedures performed on the animal models were approved by the Animal Care and Ethics Committee of POSTECH, and experiments were conducted in accordance with the approved guidelines.

**Table 2:** The experimental conditions tested in this study according to injection conditions.

	Total flow(*mL*)	Flow rate (*mL/min*)	Injection time
Single shot	0.5	6	5 *s*
	0.5	6	5 *s*
Continuous shot	0.5	0.025	5 *min*
	0.5	0.1	20 *min*

### Determination of WF and RCS

Each X-ray image contains all information along the X-ray beam propagation. Therefore, flat field correction (FFC) as an additional digital image processing technique was employed to enhance image quality^43^. The FFC is expressed as

11 $$FFC\,image=\frac{object\,image-offset\,image}{gain\,image-offset\,image}$$

where the gain image is the image captured without a test sample under the same experimental condition, and the offset image is the image taken without X-ray exposure. 10 gain images were statistically averaged. The slight movements of test samples during experiments were compensated by adopting a digital image processing technique. The WFs in each case were obtained from intensity profiles extracted along the vertical and horizontal lines (Fig. 4a and b). The thickness of the working fluid at an arbitrary position *x* can be estimated by using the Beer–Lambert law^44^;

12 $$I={I}_{0}\exp (-\mu x)$$

where *μ* is the linear absorption coefficient of the drug solution. *I*_*0*_ and *I* are the incident and transmitted intensities of X-ray beam, respectively. These estimation procedures are well described in our previous study^43^.
